# Fine‐tuning of dual‐SMAD inhibition to differentiate human pluripotent stem cells into neural crest stem cells

**DOI:** 10.1111/cpr.13103

**Published:** 2021-07-29

**Authors:** Hyun‐Mun Kim, Hye Bin Noh, Sang‐Hyuk Lee, Kun‐Gu Lee, Bomi Chang, Eunji Cheong, C. Justin Lee, Dong‐Youn Hwang

**Affiliations:** ^1^ Department of Biomedical Science Graduate School of CHA University Sungnam Korea; ^2^ Center for Cognition and Sociality Institute for Basic Science Daejeon Korea; ^3^ Brain Science Institute Korea Institute of Science and Technology Seoul Korea; ^4^ Department of Biotechnology, College of Life Science and Biotechnology, Translational Research Center for Protein Function Control Yonsei University Seoul Korea; ^5^ Department of Microbiology, School of Medicine CHA University Sungnam Korea

**Keywords:** BMP signalling, human pluripotent stem cells, modified dual‐SMAD inhibition, neural crest stem cells, p75high, p75low

## Abstract

**Objectives:**

The derivation of neural crest stem cells (NCSCs) from human pluripotent stem cells (hPSCs) has been commonly induced by WNT activation in combination with dual‐SMAD inhibition.

In this study, by fine‐tuning BMP signalling in the conventional dual‐SMAD inhibition, we sought to generate large numbers of NCSCs without WNT activation.

**Materials and methods:**

In the absence of WNT activation, we modulated the level of BMP signalling in the dual‐SMAD inhibition system to identify conditions that efficiently drove the differentiation of hPSCs into NCSCs. We isolated two NCSC populations separately and characterized them in terms of global gene expression profiles and differentiation ability.

**Results:**

Our modified dual‐SMAD inhibition containing a lower dose of BMP inhibitor than that of the conventional dual‐SMAD inhibition drove hPSCs into mainly NCSCs, which consisted of HNK^+^p75high and HNK^+^p75low cell populations. We showed that the p75high population formed spherical cell clumps, while the p75low cell population generated a 2D monolayer.

We detected substantial differences in gene expression profiles between the two cell groups and showed that both p75high and p75low cells differentiated into mesenchymal stem cells (MSCs), while only p75high cells had the ability to become peripheral neurons.

**Conclusions:**

This study will provide a framework for the generation and isolation of NCSC populations for effective cell therapy for peripheral neuropathies and MSC‐based cell therapy.

## Abstract 2

The derivation of NCSCs from hPSCs by a modified dual‐SMAD inhibition without WNT activation. We showed that the p75high population formed spherical cell clumps, while the p75low cell population generated a 2D monolayer. When the two groups were isolated and differentiated, both p75high and p75low cells were differentiated into MSCs, but peripheral neurons differentiated only from p75high cells.

## 1 INTRODUCTION

Human pluripotent stem cells (hPSCs), including human embryonic stem cells (hESCs) and human‐induced pluripotent stem cells (hiPSCs), are a promising source of transplantable cells for regenerative applications.

Cell replacement therapy for neurocristopathy, a group of pathologies resulting from abnormal neural crest development,^1^ requires the efficient generation and acquisition of homogenous neural crest stem cells (NCSCs). NCSCs are multipotent progenitors that originate from the neural tube in vertebrate embryos.^2^, ^3^ They migrate extensively and generate various cell types of the peripheral nervous system, smooth muscle, bone and connective tissue.

Previous studies have demonstrated that specification of the neuroectoderm from hPSCs requires the BMP, WNT and FGF pathways.^4^, ^5^, ^6^, ^7^, ^8^, ^9^, ^10^, ^11^, ^12^, ^13^, ^14^ Since first being reported in 2009, the dual‐SMAD inhibition method has been frequently used for the generation of neural precursor cells (NPCs) from hPSCs.^15^, ^16^ Dual‐SMAD inhibition often uses SB431542 and dorsomorphin (DM), which function by blocking the TGF‐β/Activin A/Nodal‐SMAD2/3 and BMP‐SMAD1/5/8 signalling pathways, respectively. Conventional SMAD inhibition drives hPSCs into neural rosettes, which largely consist of SOX1^+^ and PAX6^+^ NPCs; NCSCs are also found as a small population and can be isolated by cell‐sorting techniques.^17^

In this study, we describe the efficient generation of NCSCs from hPSCs by fine‐tuning BMP signalling of the conventional dual‐SMAD inhibition system commonly used for NPC generation. We detected two populations of p75‐positive NCSCs, p75high and p75low cells and showed that their differentiation abilities were different. Furthermore, we introduced a convenient method to isolate p75high cells that were free from p75low cells.

This study offers insight into the generation of NCSC populations for effective cell therapy for peripheral neuropathies and mesenchymal stem cell (MSC)‐based cell therapy.

## 2 MATERIALS AND METHODS

### 2.1 hPSC culture

H9 hESCs (WiCell Research Institute, Madison, WI, USA, Passages 45‐60) were cultured on Matrigel‐coated culture dishes (BD Biosciences, San Jose, CA, USA) with TeSR™‐E8™ medium (STEMcell Technologies, Vancouver, BC, Canada). Cells were passaged every 5 to 7 days by treatment with 0.5 mmol/L EDTA (Invitrogen, Waltham, MA, USA) for 3 minutes at 37°C and were transferred at a 1:20 ratio onto Matrigel‐coated plates with TeSR™‐E8™ medium containing 10 μmol/L Y‐27632 (Sigma‐Aldrich, St. Louis, MO, USA). Two days after passage, the medium was changed daily with fresh TeSR™‐E8™ medium without Y‐27632.

### 2.2 EB formation and induction of NCSCs

hPSCs were detached with 2 mg/mL collagenase, type IV (Worthington Biochemical Corporation, Lakewood, NJ, USA), for 30 minutes at 37°C, followed by centrifugation at 300 *g* for 5 minutes, and the suspension was transferred to a fresh bacteriological petri dish. For NCSC generation, EBs were cultured for 4 days in DMEM/F12 containing 20% knockout serum replacement, 1% non‐essential amino acids and 55 μmol/L β‐mercaptoethanol (all from Invitrogen) supplemented with 10 μmol/L SB431542 (Tocris Bioscience, Bristol, UK) and 0.5‐5 μmol/L DMH1 (or DM) (Merck Millipore, Burlington, MA, USA). During differentiation, the medium was changed daily. On day 4, EBs were attached to Matrigel‐coated dishes in NCSC differentiation medium containing 1% N2 supplement (Invitrogen), 20 ng/mL bFGF (CHA Biotech, Pangyo, Korea) and 25 μg/mL human insulin solution (Sigma‐Aldrich) and continued to differentiate for 5 more days with the medium changed every day.

### 2.3 FACS analysis and purification of NCSCs

Cells were dissociated by treatment with Accutase (Invitrogen), resuspended in 1% bovine serum albumin (BSA) (Sigma‐Aldrich) in phosphate‐buffered saline (PBS) and incubated for 15 minutes at 4°C with anti‐CD271 (p75NTR)‐PE, anti‐SOX1‐PE, anti‐CD44‐APC, anti‐CD73‐PE or anti‐CD105‐APC (all from Miltenyi Biotec, Bergisch Gladbach, Germany). The cells were washed once in 1% BSA in PBS and analysed using a BD FACSCalibur (BD Biosciences). Isotype control antibodies (Miltenyi Biotec) were used as negative controls. p75high and p75low cells were separately sorted using BD FACSAria™ III Cell Sorter (BD Biosciences).

### 2.4 Quantitative reverse transcriptase‐polymerase chain reaction (qRT‐PCR)

The total RNA samples were purified using the NucleoSpin RNA II kit (MACHEREY‐NAGEL, Duren, Germany) following the manufacturer's instructions. One microgram of total RNA was reverse‐transcribed with the ReverTra Ace qPCR RT Kit (Toyobo, Osaka, Japan) according to the manufacturer's instructions. Real‐time quantitative PCR was performed with SYBR™ Select Master Mix (Applied Biosystems, Foster City, CA, USA) and analysed by a StepOnePlus™ Real‐Time PCR System (Applied Biosystems). The qRT‐PCR conditions used in this study were as follows: (1) denaturation at 95°C for 15 seconds, (2) annealing at 60°C for 30 seconds and (3) extension at 72°C for 30 seconds. These steps were repeated for 40 cycles, followed by a final extension of 10 minutes at 72°C. The primer sequences used for PCR analysis are listed in Table S1. Glyceraldehyde 3‐phosphate dehydrogenase (GAPDH) was used as a reference gene for normalization.

### 2.5 Microarray analysis

Total RNA was purified using the NucleoSpin RNA II kit (MACHEREY‐NAGEL) according to the manufacturer's suggestions. One microgram of total RNA was utilized for global gene expression profiling using the Illumina HumanHT‐12 v4 Expression BeadChip (Illumina, San Diego, CA, USA).

### 2.6 Immunocytochemistry

Cells were fixed in 4% paraformaldehyde in PBS for 10 minutes and permeabilized with 0.2% Triton X‐100 (Sigma‐Aldrich) for 10 minutes. Cells were blocked with 5% normal goat serum, 1% BSA and 0.1% Tween‐20 (Sigma‐Aldrich) in PBS for 1 hour at RT. The cells were then incubated with primary antibodies for 1 hour at 37°C or overnight at 4°C. The primary antibodies used were for p75 (Santa Cruz Biotechnology, Dallas, TX, USA), SOX1 (R&D Systems, Minneapolis, MN, USA), TUJ1 (BioLegend, San Diego, CA, USA) and peripherin (Merck Millipore). The secondary antibodies used were conjugated with either Alexa Fluor 488 or Alexa Fluor 594 (Invitrogen). The samples were treated with 4′,6‐diamidino‐2‐henylindole (DAPI) (Sigma‐Aldrich) for 10 minutes after secondary antibody treatment. Images were captured using a Zeiss 510 fluorescein microscope (ZEISS Microscopy, Jena, Germany).

### 2.7 Mesodermal differentiation of NCSCs

For MSC generation, NCSCs were plated onto tissue culture dishes at a density of 5 X 10^4^ cell/cm^2^ in α‐MEM containing 10% FBS (all from Invitrogen). The medium was changed every 2 days, and the cells were passaged every 4‐5 days for 2 weeks.

For adipogenic differentiation, MSCs were seeded onto tissue culture dishes at a density of 2 × 10^4^ cell/cm^2^ and cultured in α‐MEM containing 10% FBS, 10 μg/mL insulin (Sigma‐Aldrich), 0.5 mmol/L 2‐isobutyl‐1‐methylxanthine (IBMX) (Sigma‐Aldrich), 20 μmol/L indomethacin (Sigma‐Aldrich) and 1 μmol/L dexamethasone (Sigma‐Aldrich) for 2 weeks with medium changes every 3 days. After induction, the cells were fixed with 10% formalin (Sigma‐Aldrich) for 30 minutes at RT, followed by incubation for 30 minutes in 0.3% Oil Red O solution (Sigma‐Aldrich).

For osteogenic differentiation, MSCs were seeded at a density of 5 × 10^3^/cm^2^ and cultured in α‐MEM containing 10% FBS, 10 nmol/L dexamethasone, 50 μmol/L ascorbic acid (Sigma‐Aldrich) and 10 mmol/L glycerol‐2‐phosphate (Sigma‐Aldrich) for 3 weeks with medium changes every 3 days. Osteogenic differentiation was confirmed with alizarin red staining. Briefly, the cells were fixed for 10 minutes at RT in 100% methanol, followed by staining with alizarin red S solution (40 mmol/L, pH 4.2) (Sigma‐Aldrich) for 10 minutes at RT.

For chondrogenic differentiation, MSCs were plated at 2 × 10^5^ cells/mL in chondrogenic medium consisting of high‐glucose DMEM (Invitrogen) supplemented with 10 ng/mL TGFβ3 (PeproTech, Rocky Hill, CT, USA), 500 ng/mL BMP‐6 (PeproTech), 50 μg/mL ascorbic acid, 50 mg/mL ITS (Sigma‐Aldrich), 40 μg/mL l‐proline (Sigma‐Aldrich), 100 μg/mL pyruvic acid (Sigma‐Aldrich) and 0.1 μmol/L dexamethasone. The medium was changed every 3 days for 4 weeks. After induction, the cells were fixed for 30 minutes at RT in 10% formalin and then stained overnight with alcian blue solution (Sigma‐Aldrich) at RT.

### 2.8 Peripheral neuronal differentiation

NCSCs were cultured on polyornithine‐laminin‐fibronectin‐coated culture dishes in DMEM/F12 medium supplemented with 1× N2, 10 ng/mL brain‐derived neurotrophic factor (BDNF) (PeproTech), 10 ng/mL glial cell‐derived neurotrophic factor (GDNF) (PeproTech), 10 ng/mL nerve growth factor (NGF) (PeproTech), 200 μmol/L ascorbic acid and 0.1 mmol/L dibutyryl cyclic AMP (dbcAMP) (all from Sigma‐Aldrich) for 10‐14 days. The medium was changed every other day and assayed by immunocytochemistry.

### 2.9 Colony‐forming unit assay of MSCs

MSCs at passage 3 were seeded into 100 mm dish at a density of 3 × 10^2^ cells in α‐MEM containing 10% FBS (all from Invitrogen). The medium was changed every 2 days. After 14 days, the cells were washed with PBS, were fixed in 4% paraformaldehyde/PBS for 10 minutes and then were stained with 3% crystal violet in 100% methanol for 5 minutes at RT.

### 2.10 Electrophysiology

Neurons cultured on individual coverslips were transferred to the recording chamber mounted on the stage of an upright microscope (Olympus, Tokyo, Japan) fitted with a 40× water‐immersion objective lens. Cells were visualized either directly via the optical microscope or indirectly via a high‐resolution CCD camera system (Orca Flash 2.1, Hamamatsu photonics, Iwata City, Japan).

Recordings were obtained using a Multiclamp 700A amplifier (Molecular Devices, Sunnyvale, CA) and were filtered at 2 kHz. Current recordings under ramp protocol and step were digitized at 10 kHz with DigiDATA 1550B1 and analysed using pCLAMP 10 software (all from Molecular Devices).

During recordings, cells were superfused at 1‐3 mL/min with external solution contained 150 mmol/L NaCl, 10 mmol/L HEPES, 3 mmol/L KCl, 2 mmol/L CaCl_2_, 2 mmol/L MgCl_2_, 5.5 mmol/L glucose, 20 mmol/L sucrose, pH adjusted to pH 7.3, and micropipettes contained internal solution composed of 140 mmol/L potassium gluconate, 10 mmol/L KCl, 1 mmol/L MgCl_2_, 0.5 mmol/L EGTA, 40 mmol/L HEPES (pH 7.2 was adjusted with KOH). Series resistance was monitored throughout the experiments, and data were discarded if changes >20% were observed.

### 2.11 Statistical analysis

Statistical analysis was performed using GraphPad Prism (GraphPad software, Inc, La Jolla, CA). Student's *t* test was used to compare 2 groups, while one‐way ANOVA with Tukey's test was used to compare the multiple groups. Only *P*‐values less than 0.05 were considered statistically significant.

## 3 RESULTS

### 3.1 Determination of the fate of hPSCs into NCSCs by fine‐tuning the BMP signalling pathway

**FIGURE 1:**
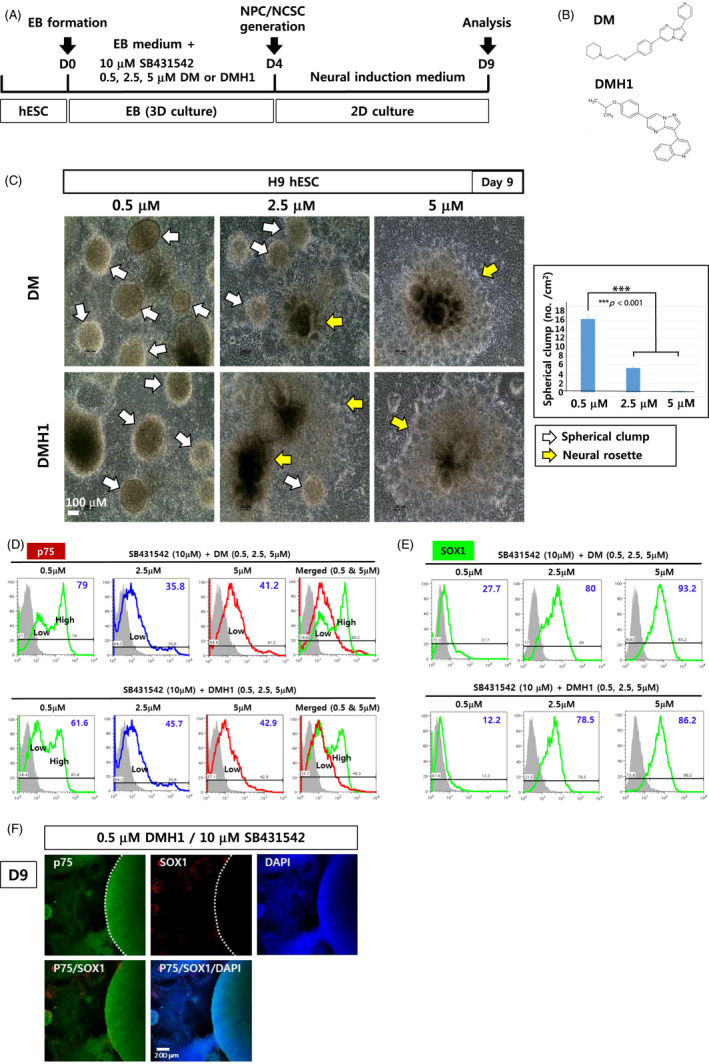
Differentiation of hPSCs into NCSCs using modified dual‐SMAD inhibition. (A) A schematic diagram summarizing the differentiation of H9 hESCs into NPCs and NCSCs. (B) Chemical structures of DM and DMH1. (C) EBs generated in suspension for 4 days in EB medium supplemented with SB431542 (10 μmol/L) and DMH1 (or DM) (0.5, 2.5 and 5 μmol/L) were then attached to dishes for another 5‐d culture. Spherical cell clumps and neural rosettes are marked by white and yellow arrows, respectively. Scale bar: 100 μm. (D, E) After 9 d of differentiation, cells were analysed with flow cytometry using antibodies against p75 (D) and SOX1 (E). (F) Immunostaining of the cells was performed with antibodies against p75 and SOX1 after differentiation with 10 μmol/L SB431542 and 0.5 μmol/L DMH1. Scale bar: 200 μm

**FIGURE 2:**
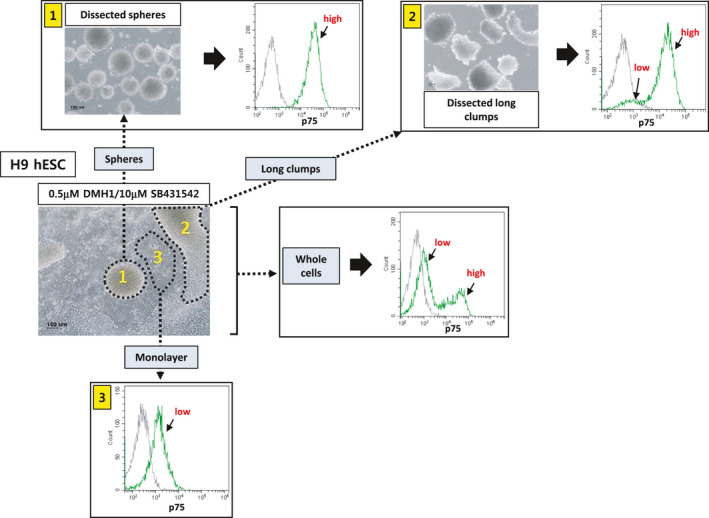
Flow cytometric analysis of the cell populations present at day 9 after differentiation. H9 hESCs were differentiated into NCSCs via EB formation using modified dual‐SMAD inhibition (0.5 μmol/L DMH1/10 μmol/L SB431542). Three different areas/structures (ie, spherical cell clumps (‘1’), non‐spherical long clumps with comet tails (‘2’) and monolayer cells (‘3’)) were dissected mechanically and subjected to flow cytometry using an antibody against p75. Independently, whole cells were also analysed by flow cytometry. Scale bar: 100 μm

The currently used dual‐SMAD inhibition approach was shown to drive hPSCs into the neuroectoderm lineage, mostly NPCs^15^, ^16^; dual‐SMAD inhibition occurs by blocking TGFβ and BMP pathways using 5‐10 μmol/L SB431542 and 5 μmol/L DM or DMH1. In our study, using H9 hESC, we noticed that decreasing the concentration of DM or DMH1 to 0.5 μmol/L dramatically changed the structure of the cell clumps (Figure 1A‐C): neural rosette structures that were formed at 5 μmol/L DM were replaced by spherical cell clumps (Figure 2, ‘1’) and their derivatives with comet tails (Figure 2, ‘2’). At 0.5 μmol/L DM (DMH1)/10 μmol/L SB431542, cells migrated away from the EBs that were attached to the dish at D4 post‐differentiation and formed spherical cell clumps (Figure 1C, *left panels*; Figure S1, *left movie clip*). In contrast, at 5 μmol/L DM (DMH1)/10 μmol/L SB431542, most of the attached EBs turned into neural rosette structures, with some NCSCs migrating away from the rosettes, eventually forming a cell monolayer (Figure 1C *right panels*, Figure S1, *right clip*): No spherical cell clumps were detected under this differentiation condition. Both neural rosettes and spherical cell clumps were observed at 2.5 μmol/L DM (DMH1)/10 μmol/L SB431542 (Figure 1C *middle panels*). Like H9 hESCs, CHA15 hESCs were differentiated into NPCs and NCSCs by the treatments with 10 mmol/L SB431542/5 mmol/L DMH1 and 10 mmol/L SB431542 and 0.5 mmol/L DMH1, respectively (Figure S2). The NCSCs derived from H9 hESCs and CHA15 hESCs were shown to retain normal karyotypes (Figure S3).

Flow cytometric analyses showed that NCSCs (p75^+^ cells) were the major cell population when 0.5 μmol/L DM (DMH1) was used in combination with 10 μmol/L SB431542 (Figure 1D, Figure S4A). Intriguingly, two populations of p75^+^ cells, p75high and p75low cells were detected at 0.5 μmol/L DM (DMH1), while only the p75low cell population was evident at 5 μmol/L DM (DMH1) (Figure 1D, Figure S4A,B). Both p75high and p75low cell populations were also HNK1^+^, another typical marker of NCSCs, suggesting that both p75^+^ cell populations were NCSCs (Figure S4A,B).

A smaller population of the cells (approximately 12.2%‐27.7%) were SOX1^+^ NPCs at 0.5 μmol/L DM (DMH1)/10 μmol/L SB431542 (Figure 1E). Immunostaining of the cells treated with 0.5 μmol/L DM (DMH1)/10 μmol/L SB431542 also showed that most of the cells, especially those of spherical cell clumps, were p75^+^, while only a small number of the cells were SOX1^+^ NPCs (Figure 1F).

Next, we further tested several low concentrations of DMH1 (0.1‐0.5 μmol/L) and found that 0.5 μmol/L DMH1 generated more spherical cell clumps and elicited better separation of the two p75^+^ cell populations than lower DMH1 concentrations (0.1, 0.2, 0.3 and 0.4 μmol/L) (Figure S5).

In summary, our results showed that dual‐SMAD inhibition drives hPSCs into mostly HNK^+^p75^+^ NCSCs or SOX1^+^ NPCs when the BMP level is high or low, respectively.

### 3.2 Spherical cell clumps consisted of p75high NCSCs

To examine the characteristics of the cells comprising the spherical cell clumps, we mechanically isolated the spheres and dissociated them into single cells for flow cytometric analysis. Our results showed that the spherical cell clumps mostly consisted of p75high cells (‘1’ in Figure 2).

In addition, we mechanically dissected non‐spherical (long) cell clumps with comet tails and subjected them to flow cytometry. The long cell clumps consisted of a mixture of p75high and p75low cells (‘2’ in Figure 2). Finally, we showed that monolayer cells around the cell clumps were mostly p75low cells (‘3’ in Figure 2).

The results indicated that p75high and p75low cells generated from dual‐SMAD inhibition with a low BMP inhibitor (0.5 μmol/L DMH1) consisted of at least two cell populations, p75high and p75low, which comprised spherical cell clumps and monolayers, respectively.

### 3.3 Gene expression profiles of the p75high and p75low cell populations

**FIGURE 3:**
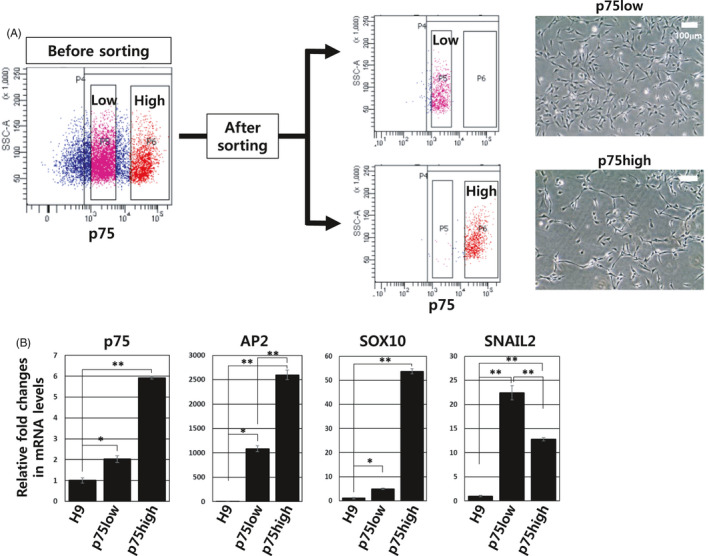
Sorting and characterization of p75high and p75low populations. (A) Two p75^+^ populations, p75high and p75low cells, were separately isolated by FACS. The cell morphology of the two cell groups is shown (*right panels*). (B) Expression levels of representative neural crest genes, p75, AP2, SOX10 and SNAIL2, were analysed using quantitative RT‐PCR. The glyceraldehyde 3‐phosphate dehydrogenase *(GAPDH)* gene was used as a normalization control. The expression level of each gene in H9 hESCs was arbitrarily set to 1. *P < .05, **P < .01

To examine the p75high and p75low cell populations, we isolated each cell population via fluorescence‐activated cell sorting (FACS) using an antibody against p75 (Figure 3A). No significant difference was detected in cell morphology between the two groups (Figure 3A, *right panels*). Quantitative RT‐PCR analysis showed that typical markers for NCSCs, p75, AP2 and SOX10 were expressed more abundantly in the p75high population than in the p75low population (Figure 3B). Noticeably, Snail2, another neural crest marker that is implicated in epithelial‐mesenchymal transitions, was expressed more significantly in the p75low cell population than in the p75high cell population (Figure 3B).

**FIGURE 4:**
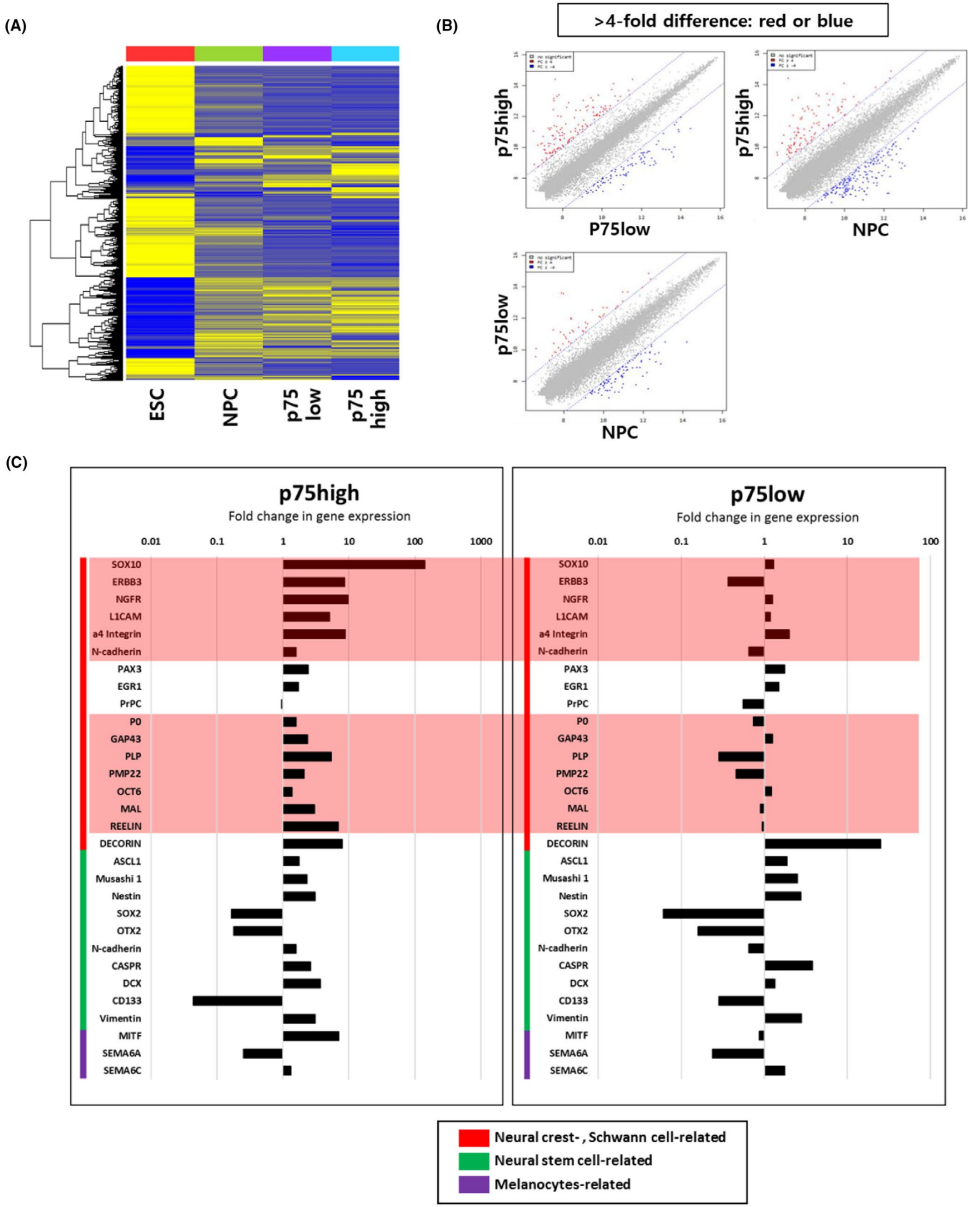
Global gene expression analysis of p75high and p75low cell populations. (A) Heatmap of the microarray data from ESCs, NPCs, p75low cells and p75low cells. (B) Scatter plot of gene expression profiles for p75high cells—p75low cells, p75high cells—NPCs and p75low cells—NPCs. The genes that were differentially expressed by more than 4‐fold are indicated as red or blue dots. (C) Fold differences in genes associated with the neural crest, Schwann cells, neural stem cells and melanocytes were compared between p75high and p75low cells. All values are relative to undifferentiated hESC. The genes that are highly differentially expressed between p75high and p75low cells are denoted in pink boxes

To compare the two p75^+^ populations more carefully, we compared the global gene expression profiles of the H9 hESCs, NPCs, p75high cell group and p75low cell group (Figure 4). Heatmap analysis showed that substantial differences in global gene expression were evident between the two p75^+^ cell groups (Figure 4A). The scatter plot indicated gene probes that were more than 4‐fold different among the NPCs, p75high and p75low cell groups (Figure 4B). Intriguingly, neural crest‐ or Schwann cell‐related genes were more abundantly expressed in the p75high population than in the p75low population, while no dramatic change in the expression of neural stem cell‐ and melanocyte‐related genes was detected between the two groups (Figure 4C). The list of genes that were differentially expressed by more than 4‐fold between p75high and p75low cells are shown in Table S2: neural crest‐, peripheral nervous system‐ or Schwann cell‐related genes, such as SOX10 (SRY‐box transcription factor 10), ERBB3 (Erb‐b2 receptor tyrosine kinase 3), INSC (protein inscuteable homolog), EDNRB (endothelin receptor type B), GFRA2 (GDNF family receptor alpha‐2), MEGF10 (multiple epidermal growth factor‐like domains protein 10), PLP1 (myelin proteolipid protein 1), L1CAM (neural cell adhesion molecule L1) and TFAP2C (transcription factor AP‐2 gamma), were much more highly expressed in p75high cells than in p75low cells.

Taken together, these results showed that p75high cells expressed more neural crest‐related genes than p75low cells.

### 3.4 Differentiation of p75low and p75high cells into mesenchymal stem cells

**FIGURE 5:**
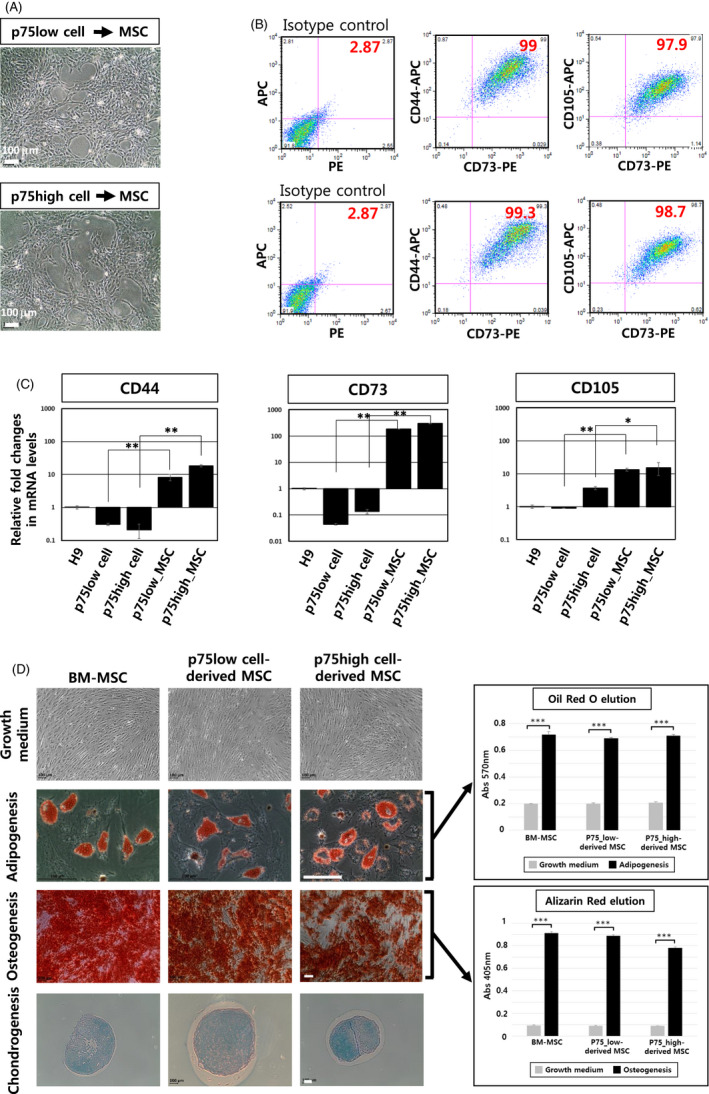
Mesodermal differentiation of hESC‐derived p75high and p75low cells. (A) Bright‐field images of MSCs derived from FACS‐sorted p75high and p75low cells are shown. Scale bar: 100 μm. (B) Flow cytometric analysis of MSCs for the representative MSC surface markers CD44, CD73 and CD105 (*top panels*: p75low cell‐derived MSCs, *bottom panels*: p75high cell‐derived MSCs). (C) Real‐time RT‐PCR data on the expression of three MSC markers in MSCs derived from p75high cells and p75low cells. *GAPDH* gene was used as a normalization control. *P < .05, **P < .01. (D) p75high cell‐derived and p75low cell‐derived MSCs were differentiated into adipocytes, osteocytes and chondrocytes. Bone marrow (BM)‐MSCs were used as a positive control. Both adipogenesis and osteogenesis were quantified using oil red and alizarin red solutions (*right graphs*). Scale bar: 100 μm. **p < .001

NCSCs are multipotent and can differentiate into a variety of cell types from peripheral nervous system and mesodermal tissue lineages.^18^ To examine the differentiation capability of p75high and p75low cells into MSCs, cells were cultured in DMEM/F12 growth medium containing 10% foetal bovine serum (Figure 5A). No significant difference was detected in cell morphology between the two groups at 2 weeks post‐differentiation (Figure 5A). On day 14 post‐differentiation, both flow cytometric and quantitative RT‐PCR analyses showed similar expression levels of MSC markers, such as CD44, CD73 and CD105, in both p75^+^ cell population‐derived MSCs (Figure 5B,C). The NCSC‐derived MSCs displayed efficient self‐renewal ability as shown by high colony‐forming efficiency (Figure S6).

We next confirmed that MSCs derived from both p75high and p75low cell populations could be converted into adipocytes, osteocytes and chondrocytes (Figure 5D).

Together, the results showed that both the p75high and p75low populations retained the capability to become MSCs.

### 3.5 p75high cells, but not p75low cells, could be differentiated into peripheral neurons

**FIGURE 6:**
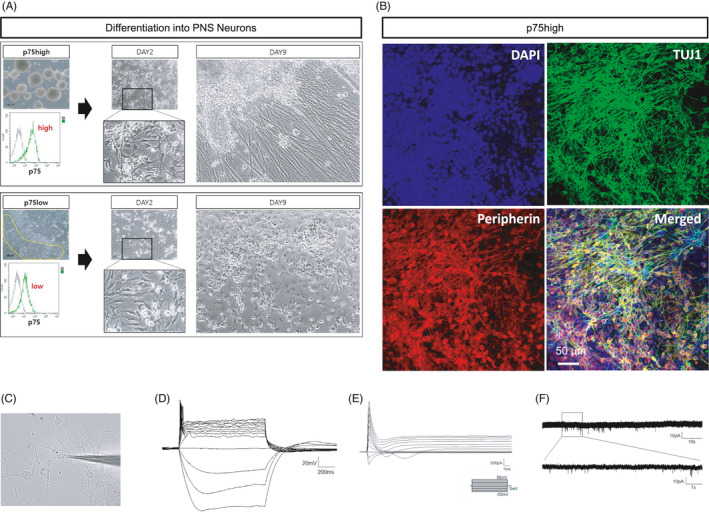
Generation of peripheral neurons from either hESC‐derived p75high or p75low cells. (A) p75high cells (*top*) and p75low cells (*bottom*) were obtained from mechanically dissected spherical cell clumps and cell monolayers, respectively. Both cell groups were induced to differentiate into neurons. (B) Neurons derived from p75high cells were immunostained for TUJ1 and peripherin. Scale bar: 50 μm. (C‐F) Electrophysiological analysis of NCSC‐derived peripheral neurons was performed. The morphology of the neurons recorded was shown (C). Representative action potential firing in the peripheral neurons was shown. Scale bar: 20 mV, 200 ms (D). Sodium channel current was shown in the neurons by voltage‐clamp step protocol. Scale bar: 500 pA, 1 ms (E). The trace of spontaneous EPSCs from the neurons was shown. Scale bar: 10 pA, 10 s (upper); 10 pA, 1 s (enlarged trace, bottom) (F)

Because gene expression profile analysis showed that p75high cells expressed neural crest‐related genes more significantly than p75low cells, we examined whether p75high cells can be differentiated into peripheral neurons more efficiently than p75low cells. To obtain p75high cells, only spherical cell clumps were mechanically obtained and confirmed, and most of the cells were p75high by flow cytometry (Figure 6A, *top*). Monolayer cells were also mechanically detached from the dish, and it was confirmed that only p75low cells were collected (Figure 6A, *bottom*). Both types of p75^+^ cells were differentiated into peripheral neurons in neuronal induction medium containing GDNF, BDNF, NGF and dbcAMP. p75high cells were differentiated into neuronal cells with processes (Figure 6A, *top*) that were stained with TUJ1 and peripherin (Figure 6B). The neurons displayed typical electrophysiological properties of neurons (Figure 6C‐F). In contrast, p75low cells mostly died during the neuronal differentiation procedure (Figure 6A, *bottom*).

These results showed that the p75high, but not p75low, cell population retained the capability to become peripheral neurons. Our results suggested that isolation of the p75high cell population would be required to develop an efficient cell therapy for peripheral neuropathies.

## 4 DISCUSSION

Early studies have reported the differentiation of hPSCs into NCSCs by coculture with the mouse stromal cell lines PA6 and MS5.^17^, ^19^ Later, a dual‐SMAD inhibition method was developed to drive the differentiation of hPSCs into the neuroectoderm lineage.^15^, ^16^ However, dual‐SMAD inhibition generates mostly neural rosettes, which mainly consist of NPCs with some p75^+^ NCSCs in the periphery of the neural rosettes.^15^, ^20^ To direct the differentiation of hPSCs towards NCSCs, WNT signalling activation was performed along with dual‐SMAD inhibition.^21^, ^22^, ^23^ In contrast, some studies used inhibition of only TGFβ/Activin A/Nodal signalling, not BMP signalling, in combination with WNT activation.^24^, ^25^ Most studies used glycogen synthase kinase 3 (GSK3) inhibitors, such as CHIR99021 and 6‐bromoindirubin‐3′‐oxime (BIO), to activate Wnt pathway (21‐25). Instead of GSK3 inhibitors, the addition of Wnt3a protein also induced NCSC specification from hPSCs, suggesting that Wnt3a was one of the Wnt family proteins involved in this process.^22^, ^24^ Although the implication of Wnt pathway in NCSC specification was well established, the role of BMP signalling modulation in NCSC derivation from hPSCs has yet to be clarified.

In this study, we used only dual‐SMAD inhibition (ie, inhibition of both BMP and TGFβ/Activin A/Nodal signalling) without WNT activation to differentiate hPSCs into NCSCs. We fixed the concentration of SB431542 at 10 μmol/L to inhibit TGFβ/Activin A/Nodal signalling and tested various concentrations (0.5, 2.5 and 5 μmol/L) of DMH1, a BMP signalling inhibitor, for the generation of NCSCs: 10 μmol/L was the most widely used concentration of SB431542 in dual‐SMAD inhibition.^22^, ^26^, ^27^ We found that hPSCs differentiated mostly into p75^+^ NCSCs at 0.5 μmol/L DMH1/10 μmol/L SB431542, while mostly SOX1^+^ NPCs were generated at 5 μmol/L DMH1/10 μmol/L SB431542. Intriguingly, two p75^+^ cell populations, p75high and p75low, were detected at 10 μmol/L SB431542/0.5 μmol/L DMH1. The presence of p75high and p75low populations has been previously reported, although the differences between these two populations have not been investigated thoroughly.^23^, ^24^, ^25^ Strikingly, we noticed the formation of spherical cell clumps that consisted of p75high cells. Although these spherical cell clumps have been reported previously in one study (named ‘dense aggregates’), no detailed characterization of these spheres has been performed, especially in relation to p75high and p75low cell populations.^28^ Our study first reported that spherical cell clumps were composed of p75high cells only.

The percentage of p75^+^ NCSCs among total cells resulting from our differentiation procedure was variable from experiment to experiment but was approximately 62%‐87%. The percentage of the p75high cell population among the p75^+^ NCSCs was also variable and was approximately 30%‐70% depending on experiments. The most remarkable advantage of our differentiation condition was that the p75high and p75low cell populations can be easily isolated by mechanically dissecting spherical cell clumps and monolayer cells, respectively (Figure 2).

It would be interesting to unveil the characteristics of p75high and p75low cells, especially in terms of differentiation potential. Intriguingly, both p75high and p75low cell populations could be differentiated into MSCs efficiently. No significant differences in MSC marker expression, proliferation or differentiation capability were detected between p75high NCSC‐ and p75low NCSC‐derived MSCs. In contrast, strikingly, only p75high cells were able to be differentiated into peripheral neurons, while p75low cells died during neuronal differentiation. In support of this observation, global gene expression analysis revealed that p75high cells expressed more neural crest‐ and peripheral neural‐related genes than p75high cells. To the best of our knowledge, this is the first study to demonstrate differences between p75high and p75low cell populations.

In summary, this study described a new differentiation system for the efficient induction of NCSCs from hPSCs by fine‐tuning BMP signalling in a conventional dual‐SMAD inhibition system used for NPC generation. Our results showed that at low concentrations of BMP inhibitor (0.5 μmol/L) during dual‐SMAD inhibition, neuroectodermal differentiation was geared to p75^+^ NCSC generation instead of NPC generation. Notably, our NCSC specification protocol did not use GSK3 inhibitors or Wnt3a protein. Unveiling the underlying mechanisms and potential connection between BMP and Wnt pathways in NCSC specification from hPSCs is of interest and would be the future research goal.

Furthermore, the HNK^+^p75^+^ NCSCs consisted of two subpopulations, p75high and p75low cells: p75high cells can be differentiated into mesenchymal lineage and peripheral neurons, but p75low cells can be differentiated into mesenchymal lineage only.

This study provides insight into the early differentiation of NCSCs from hPSCs and offers a useful platform for the isolation of a p75high cell population to treat many neural crest‐related diseases.

## CONFLICTS OF INTEREST

All authors of this paper declare no potential conflicts of interest.

## AUTHOR CONTRIBUTIONS

HK, HN, SL and DH designed the project. HK, HN and SL performed the development of methodology. HK, HN, SL, BC, EC, CJL and DH performed acquisition of data, analysis and interpretation and wrote the paper. HK, SL and DH performed review, and/or revision of the manuscript. KL supported technical or material support. DH performed study supervision.

## Supporting information

Figures S1‐S6

Table S1

Table S2

## Data Availability

The data that support the findings of this study are available from the corresponding author upon reasonable request.
