# Supplementary material for: Fine‐tuning of dual‐SMAD inhibition to differentiate human pluripotent stem cells into neural crest stem cells
Source: Cell Prolif. 2021 Jul 29;54(9):e13103. doi: 10.1111/cpr.13103 (PMC8450125; doi:10.1111/cpr.13103)
Supplement: Supplementary file 1 — Figures S1‐S6 [file CPR-54-e13103-s003.pptx]

## Slide 1
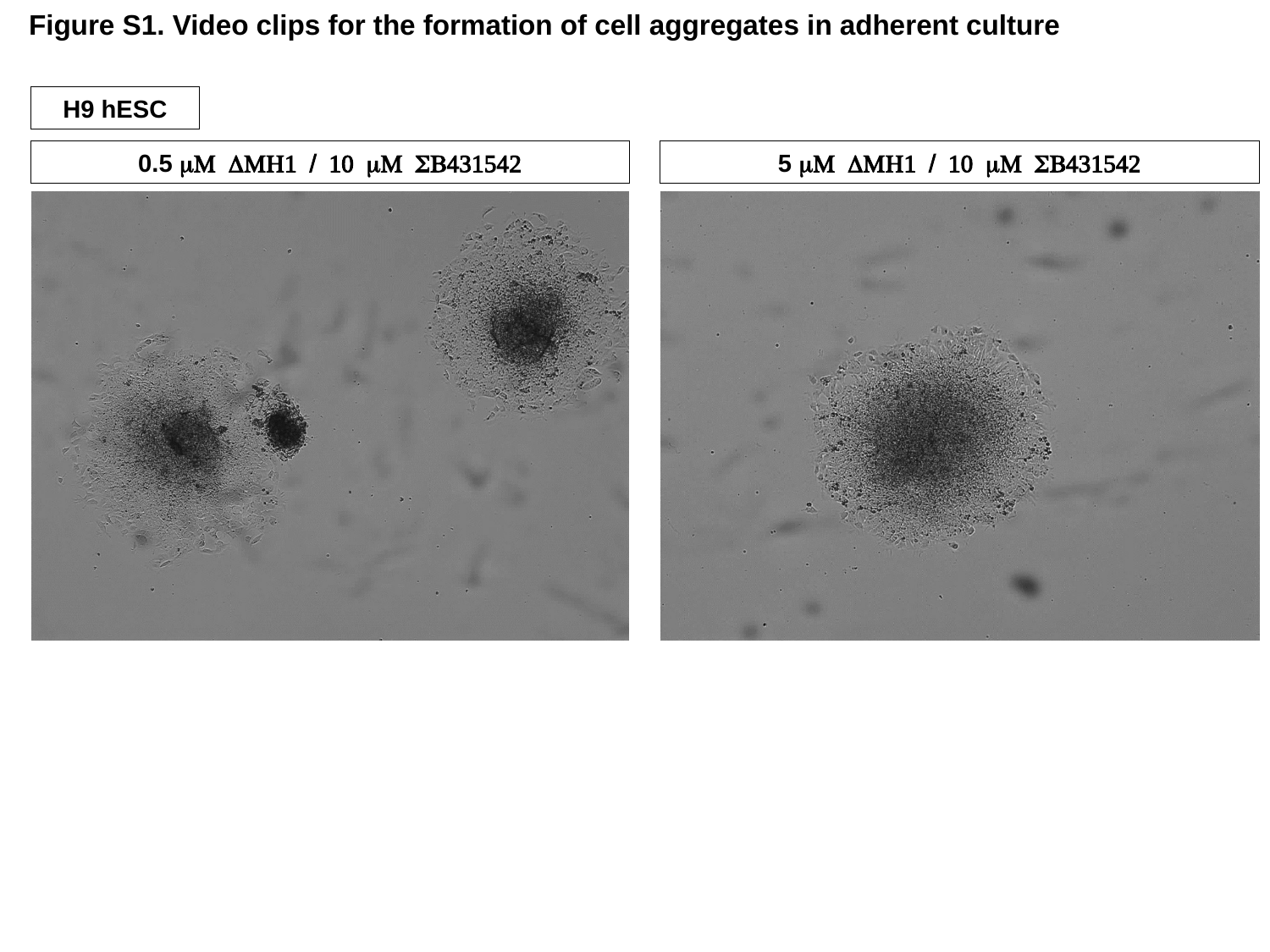

Figure S1. Video clips for the formation of cell aggregates in adherent culture
H9 hESC
0.5 mM DMH1 / 10 mM SB431542
5 mM DMH1 / 10 mM SB431542

## Slide 2
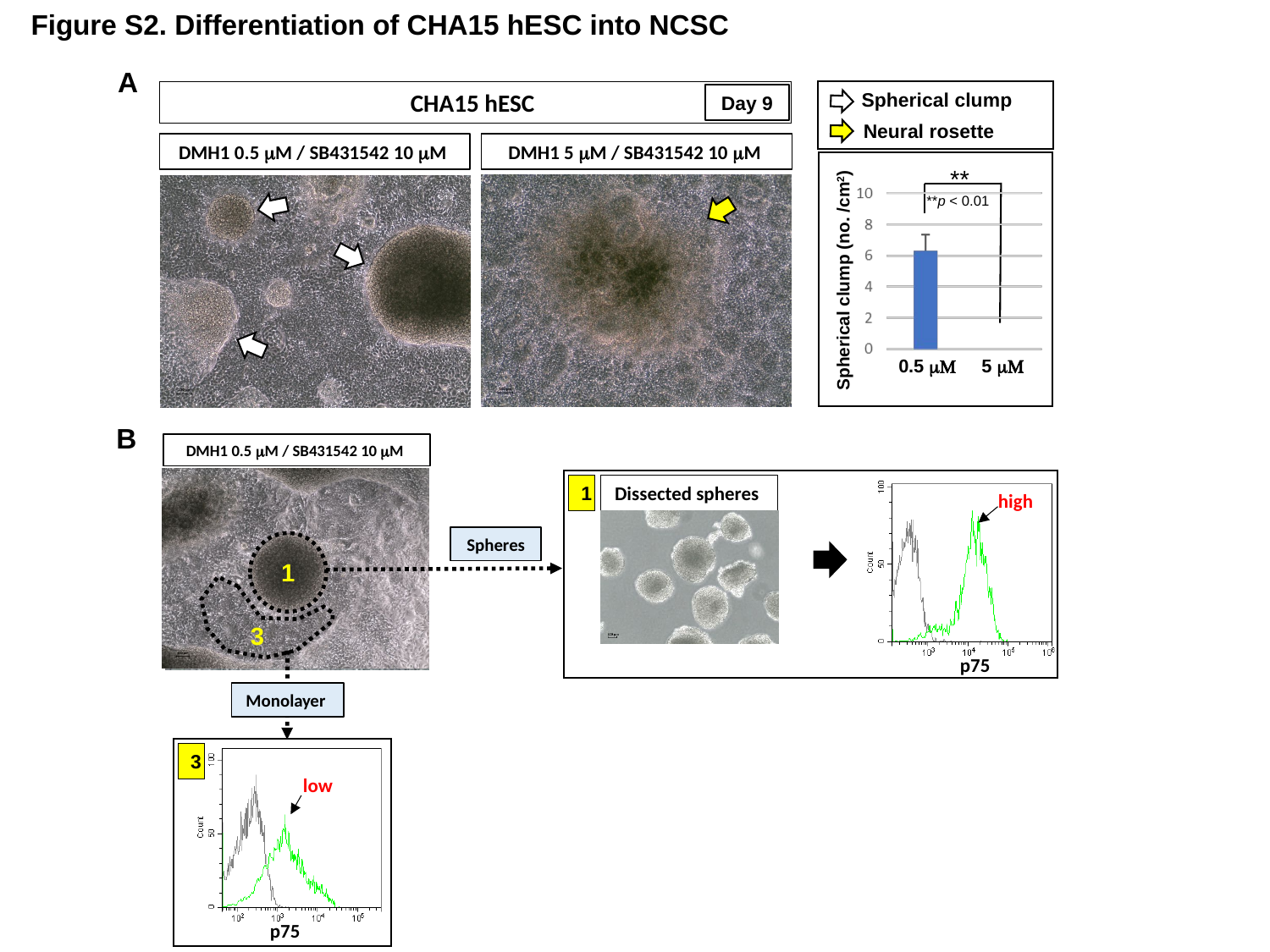

Figure S2. Differentiation of CHA15 hESC into NCSC
A
Spherical clump
Neural rosette
CHA15 hESC
Day 9
DMH1 0.5 mM / SB431542 10 mM
DMH1 5 mM / SB431542 10 mM
**
**p < 0.01
Spherical clump (no. /cm2)
5 mM
0.5 mM
B
DMH1 0.5 mM / SB431542 10 mM
1
Dissected spheres
high
p75
Spheres
1
3
Monolayer
3
low
p75

## Slide 3
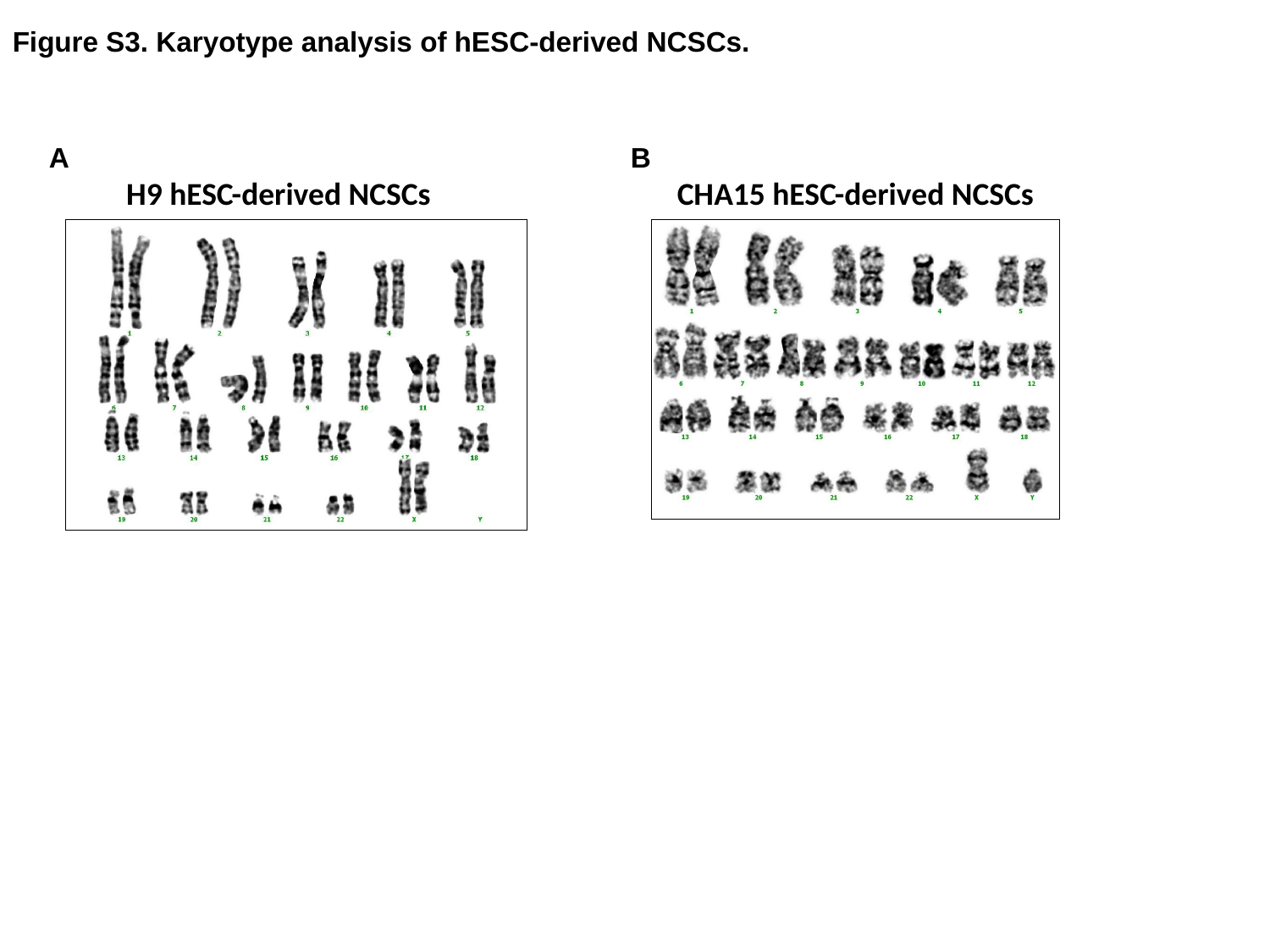

Figure S3. Karyotype analysis of hESC-derived NCSCs.
A
B
H9 hESC-derived NCSCs
CHA15 hESC-derived NCSCs

## Slide 4
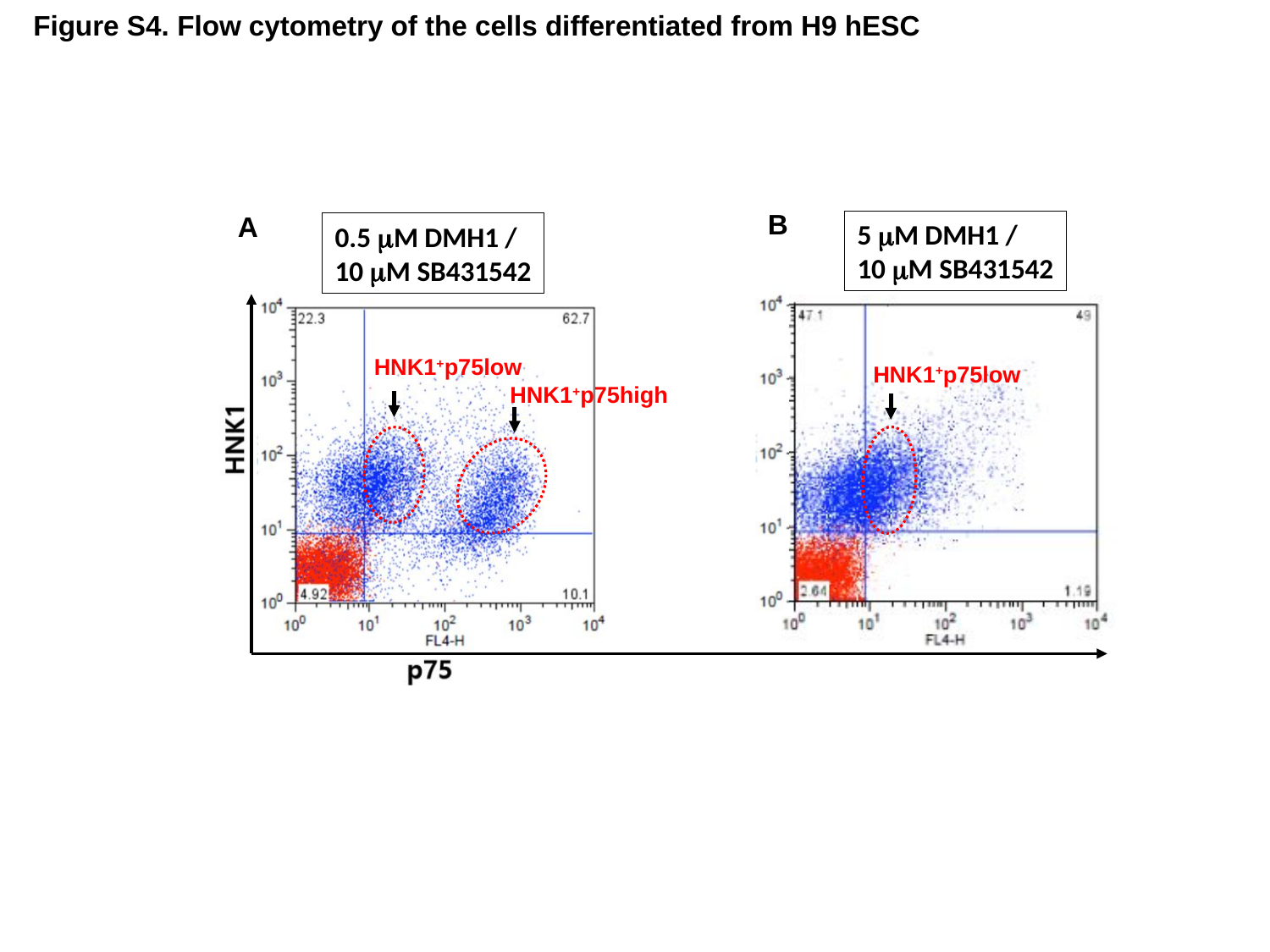

Figure S4. Flow cytometry of the cells differentiated from H9 hESC
B
A
5 mM DMH1 /
10 mM SB431542
0.5 mM DMH1 /
10 mM SB431542
HNK1+p75low
HNK1+p75low
HNK1+p75high

## Slide 5
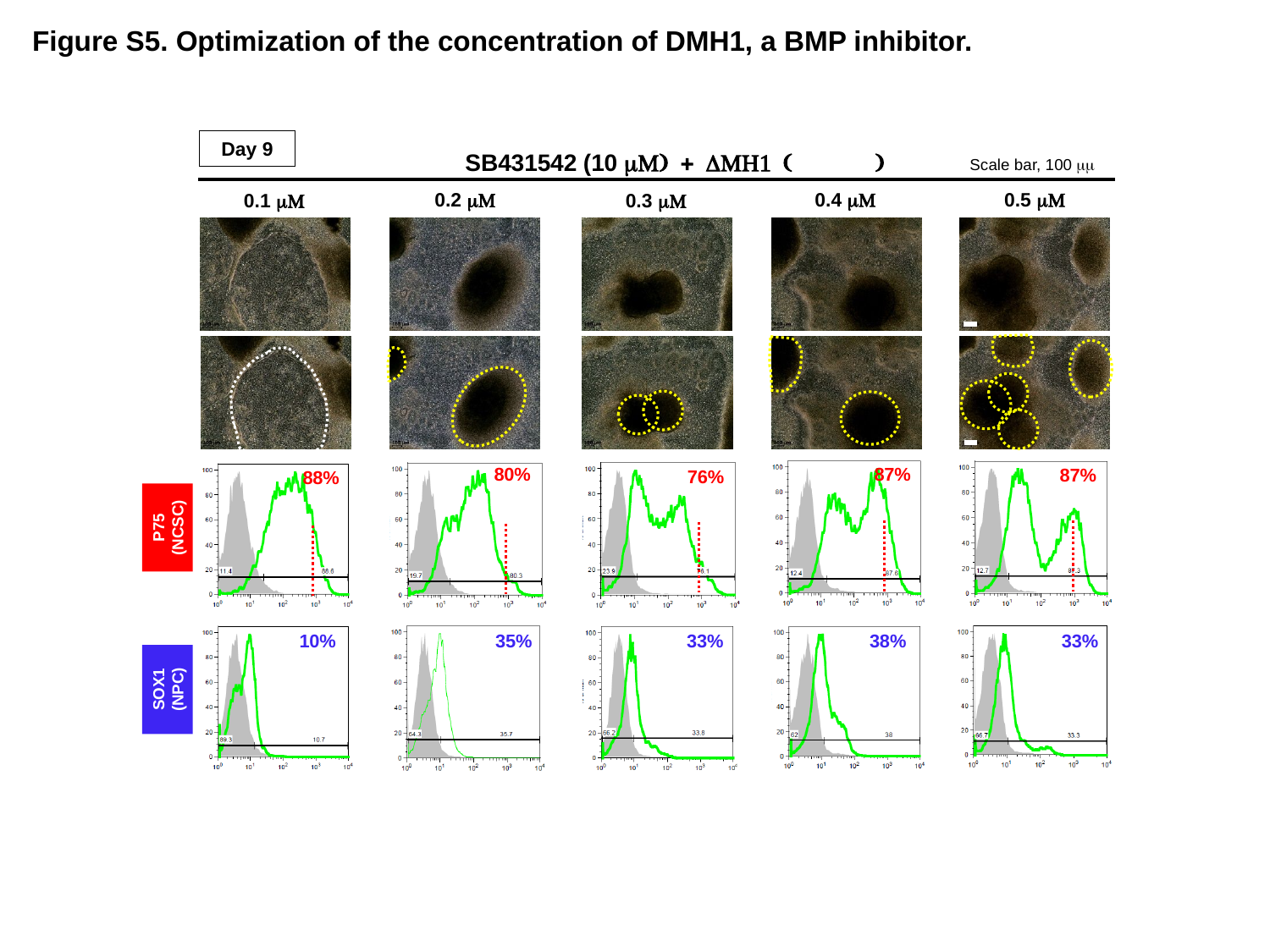

Figure S5. Optimization of the concentration of DMH1, a BMP inhibitor.
Day 9
SB431542 (10 mM) + DMH1 ( )
Scale bar, 100 mm
0.4 mM
0.5 mM
0.2 mM
0.1 mM
0.3 mM
87%
80%
87%
76%
88%
P75
(NCSC)
10%
35%
33%
38%
33%
SOX1
(NPC)

## Slide 6
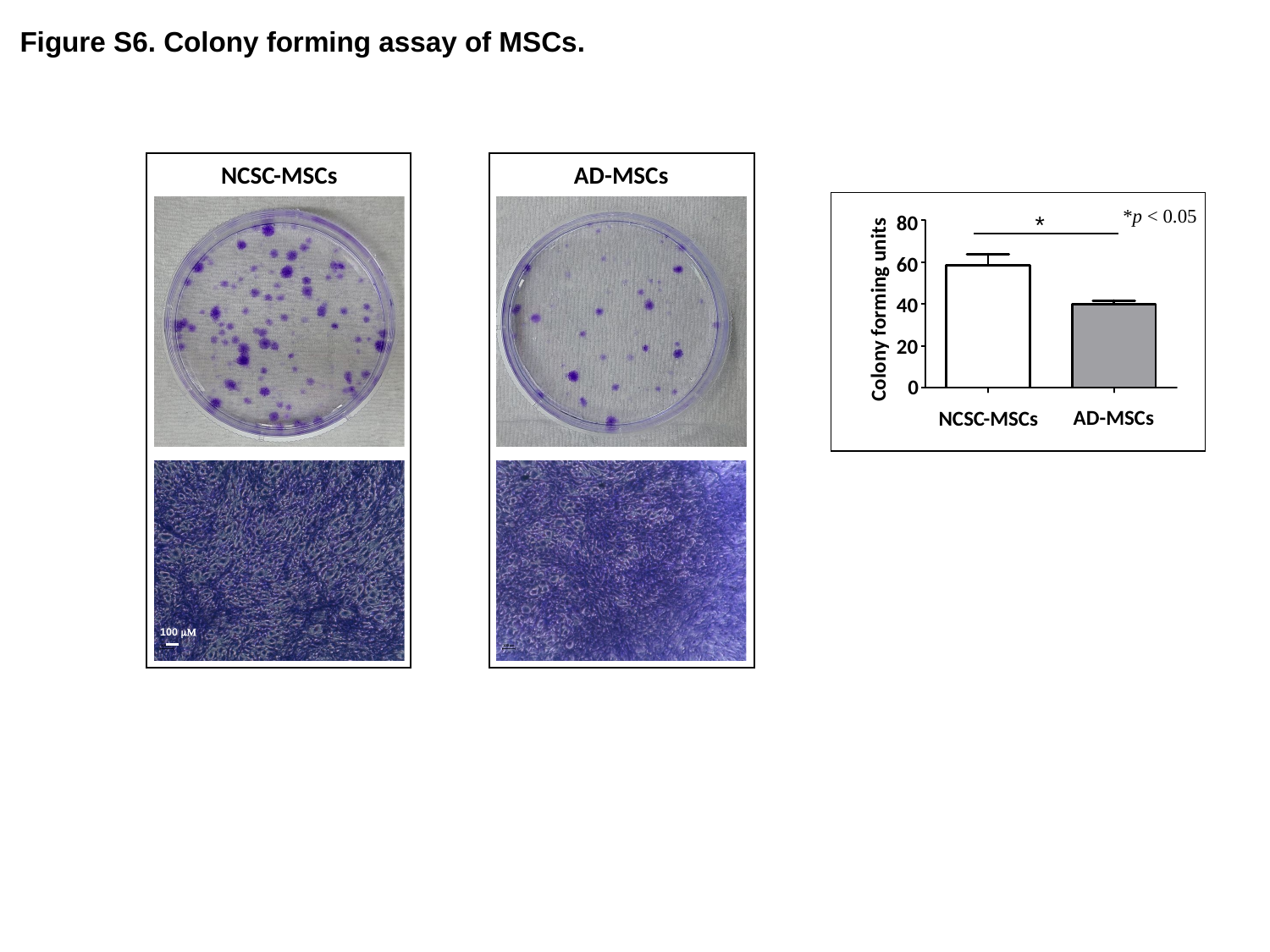

Figure S6. Colony forming assay of MSCs.
NCSC-MSCs
AD-MSCs
80
*
60
40
Colony forming units
20
0
AD-MSCs
NCSC-MSCs
*p < 0.05
100 mM

## Slide 7
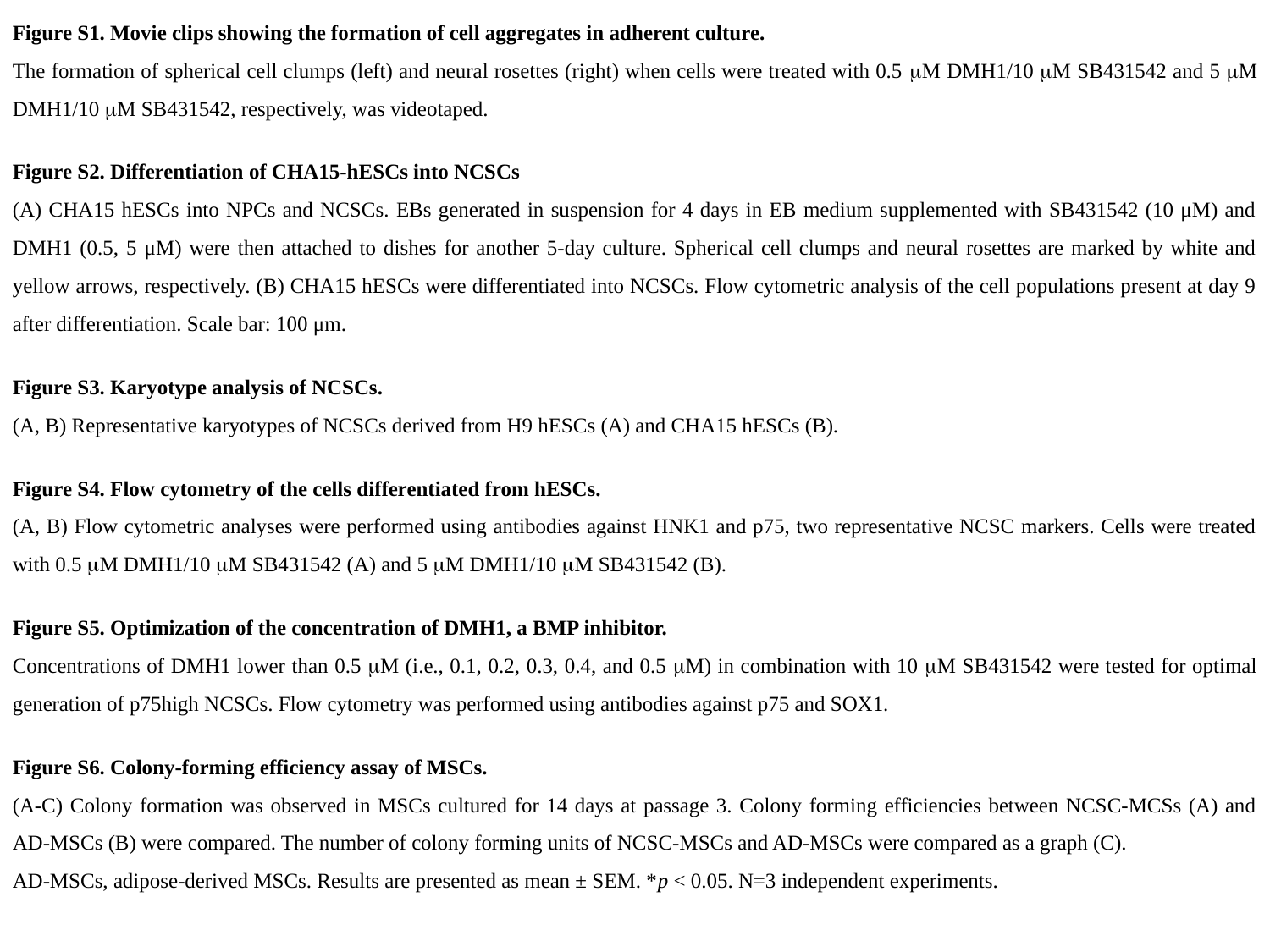

Figure S1. Movie clips showing the formation of cell aggregates in adherent culture.
The formation of spherical cell clumps (left) and neural rosettes (right) when cells were treated with 0.5 μM DMH1/10 μM SB431542 and 5 μM DMH1/10 μM SB431542, respectively, was videotaped.
Figure S2. Differentiation of CHA15-hESCs into NCSCs
(A) CHA15 hESCs into NPCs and NCSCs. EBs generated in suspension for 4 days in EB medium supplemented with SB431542 (10 μM) and DMH1 (0.5, 5 μM) were then attached to dishes for another 5-day culture. Spherical cell clumps and neural rosettes are marked by white and yellow arrows, respectively. (B) CHA15 hESCs were differentiated into NCSCs. Flow cytometric analysis of the cell populations present at day 9 after differentiation. Scale bar: 100 μm.
Figure S3. Karyotype analysis of NCSCs.
(A, B) Representative karyotypes of NCSCs derived from H9 hESCs (A) and CHA15 hESCs (B).
Figure S4. Flow cytometry of the cells differentiated from hESCs.
(A, B) Flow cytometric analyses were performed using antibodies against HNK1 and p75, two representative NCSC markers. Cells were treated with 0.5 μM DMH1/10 μM SB431542 (A) and 5 μM DMH1/10 μM SB431542 (B).
Figure S5. Optimization of the concentration of DMH1, a BMP inhibitor.
Concentrations of DMH1 lower than 0.5 μM (i.e., 0.1, 0.2, 0.3, 0.4, and 0.5 μM) in combination with 10 μM SB431542 were tested for optimal generation of p75high NCSCs. Flow cytometry was performed using antibodies against p75 and SOX1.
Figure S6. Colony-forming efficiency assay of MSCs.
(A-C) Colony formation was observed in MSCs cultured for 14 days at passage 3. Colony forming efficiencies between NCSC-MCSs (A) and AD-MSCs (B) were compared. The number of colony forming units of NCSC-MSCs and AD-MSCs were compared as a graph (C).
AD-MSCs, adipose-derived MSCs. Results are presented as mean ± SEM. *p < 0.05. N=3 independent experiments.
